# Supplementary material for: Convection-enhanced delivery of dexamethasone in glioma models suppresses myeloid inflammation while avoiding systemic toxicities
Source: J Clin Invest. 2026 Aug 18;136(19):e200550. doi: 10.1172/JCI200550 (PMC13626830; doi:10.1172/JCI200550)
Supplement: Supplementary file 1 [file jci-136-200550-s320.pdf]

A

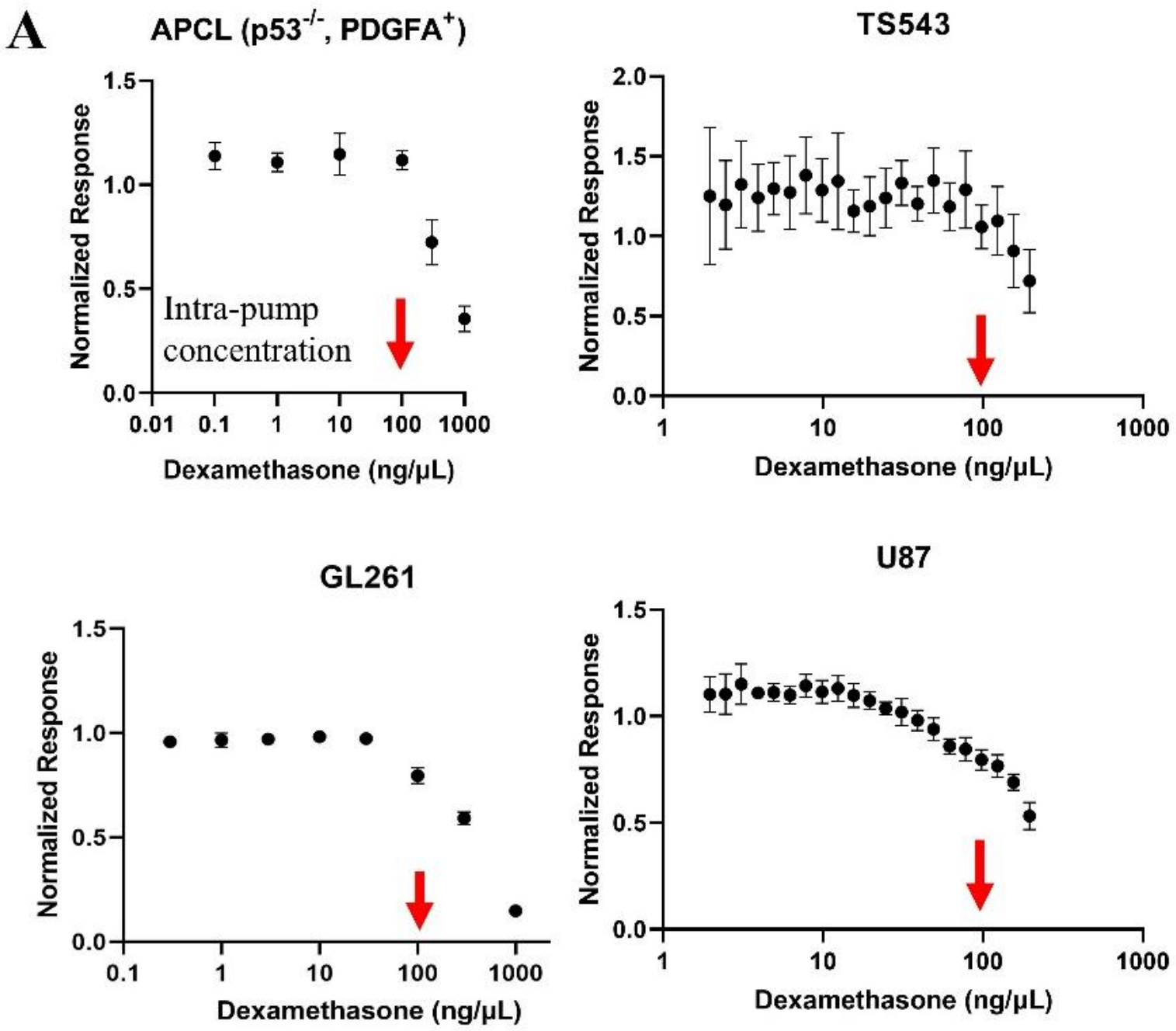

B

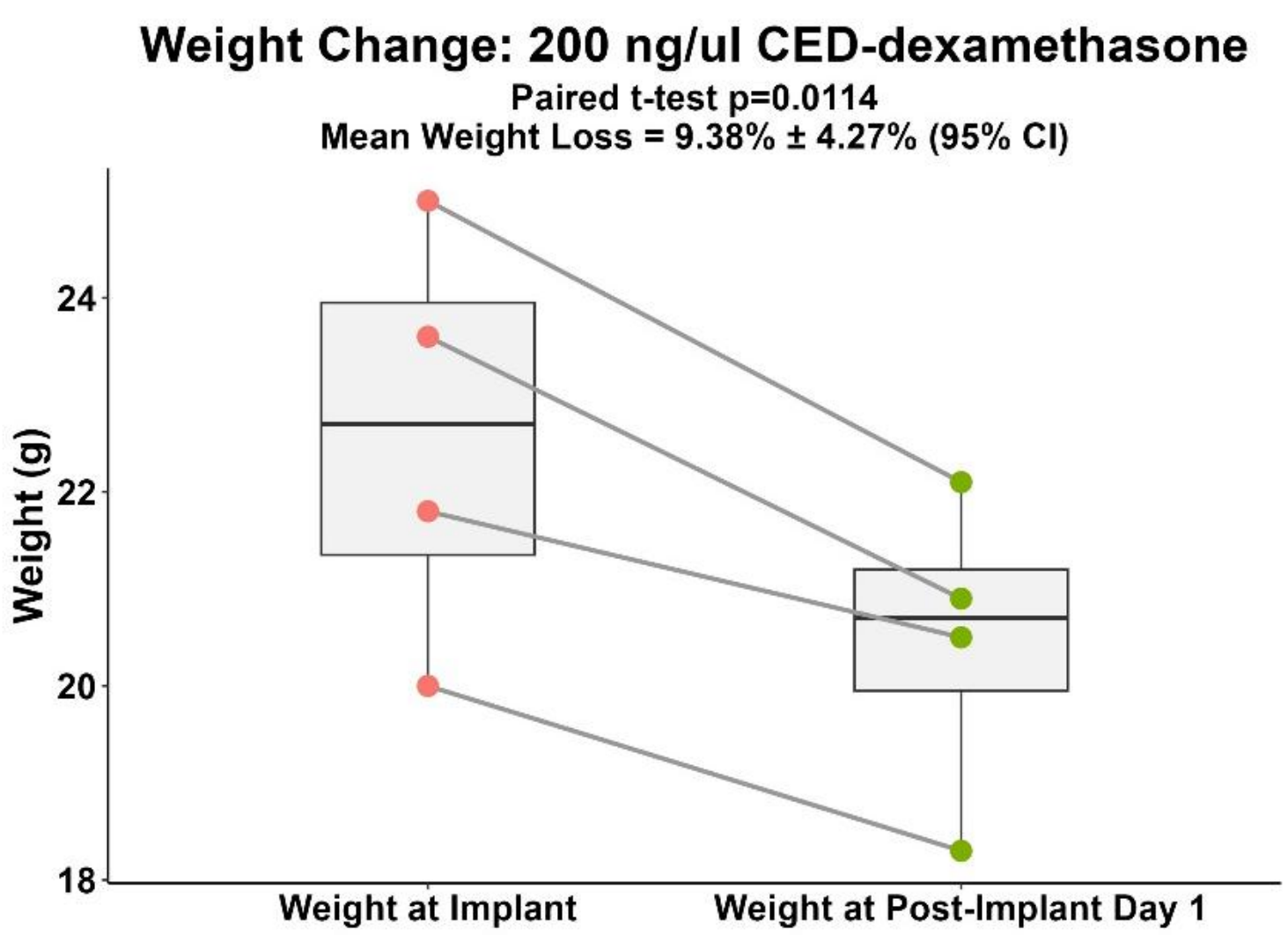

Supplementary Figure 1: **A** – The IC50 of dexamethasone was determined to be 549 ± 289 ng/µl (1,399 ± 736 µM) for murine APCL (p53$^{-/-}$, PDGFA^{+}) cells, 705 ± 219 ng/µl (1,796 ± 558 µM) in murine GL261 cells, 202 ± 19 ng/µl (515 ± 48 µM) in human TS543 glioma cells, and 194 ± 23 ng/µl (494 ± 59 µM) in human U87 cells. IC50 ± SE. **B** – Mice receiving CED-dexamethasone at a concentration of 200 ng/µl lost a significant amount of weight 1 day after pump implant (9.4% ± 4.27% (mean body weight loss ± 95% CI); paired t-test p = 0.01).

A

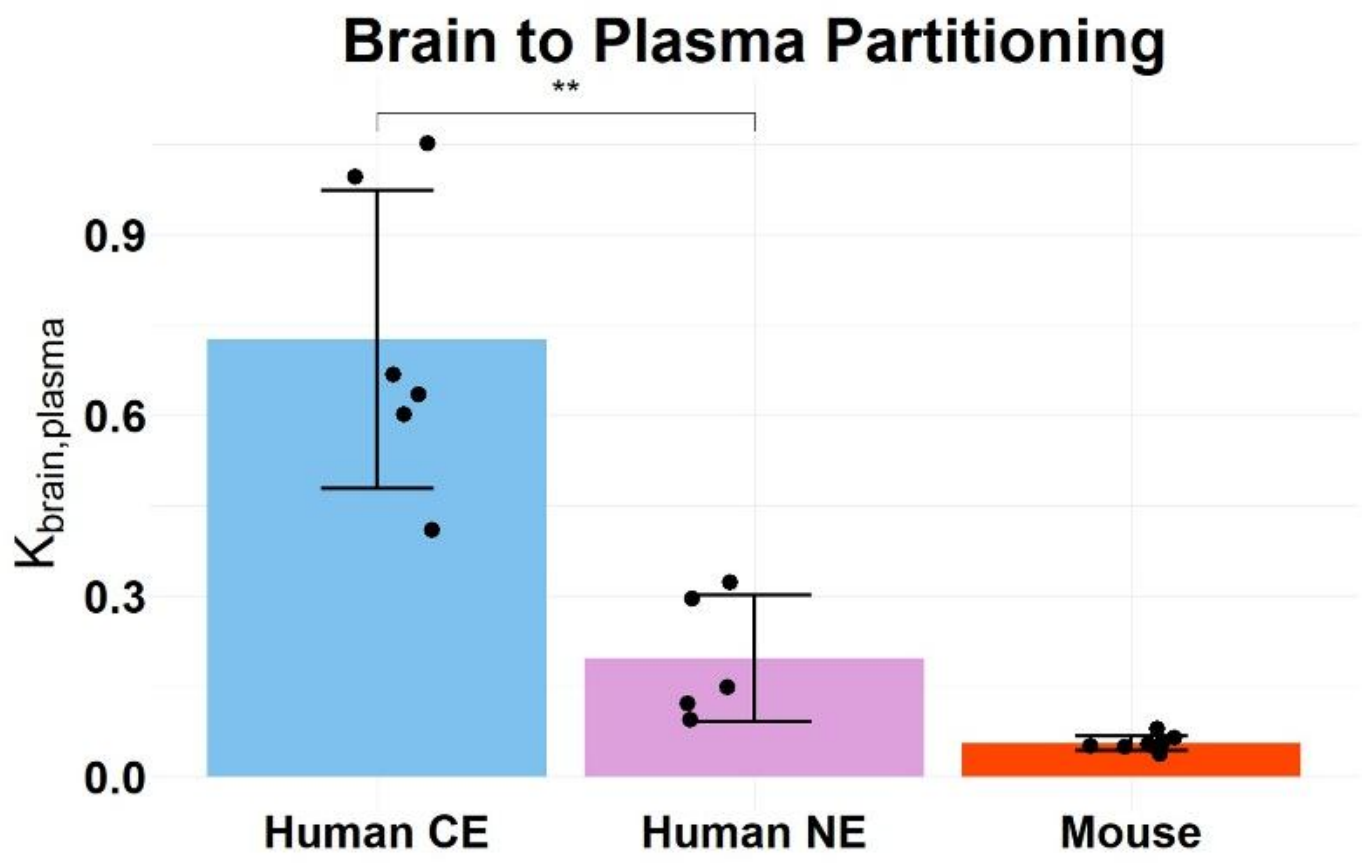

Supplementary Figure 2: **A** – The partitioning coefficient between brain and plasma in human contrast-enhancing tumor samples, non-enhancing samples, and mouse tumor-bearing brain quadrants. Contrast-enhancing tumor samples had brain to plasma concentration ratio of 73 ± 20% (mean ± 2*SE), while non-enhancing tumor samples had a brain to plasma concentration ratio of 20 ± 10% (Welch t-test, p = 0.0021). In our murine studies, the brain to plasma concentration in mice receiving systemic dexamethasone was 6 ± 2%.

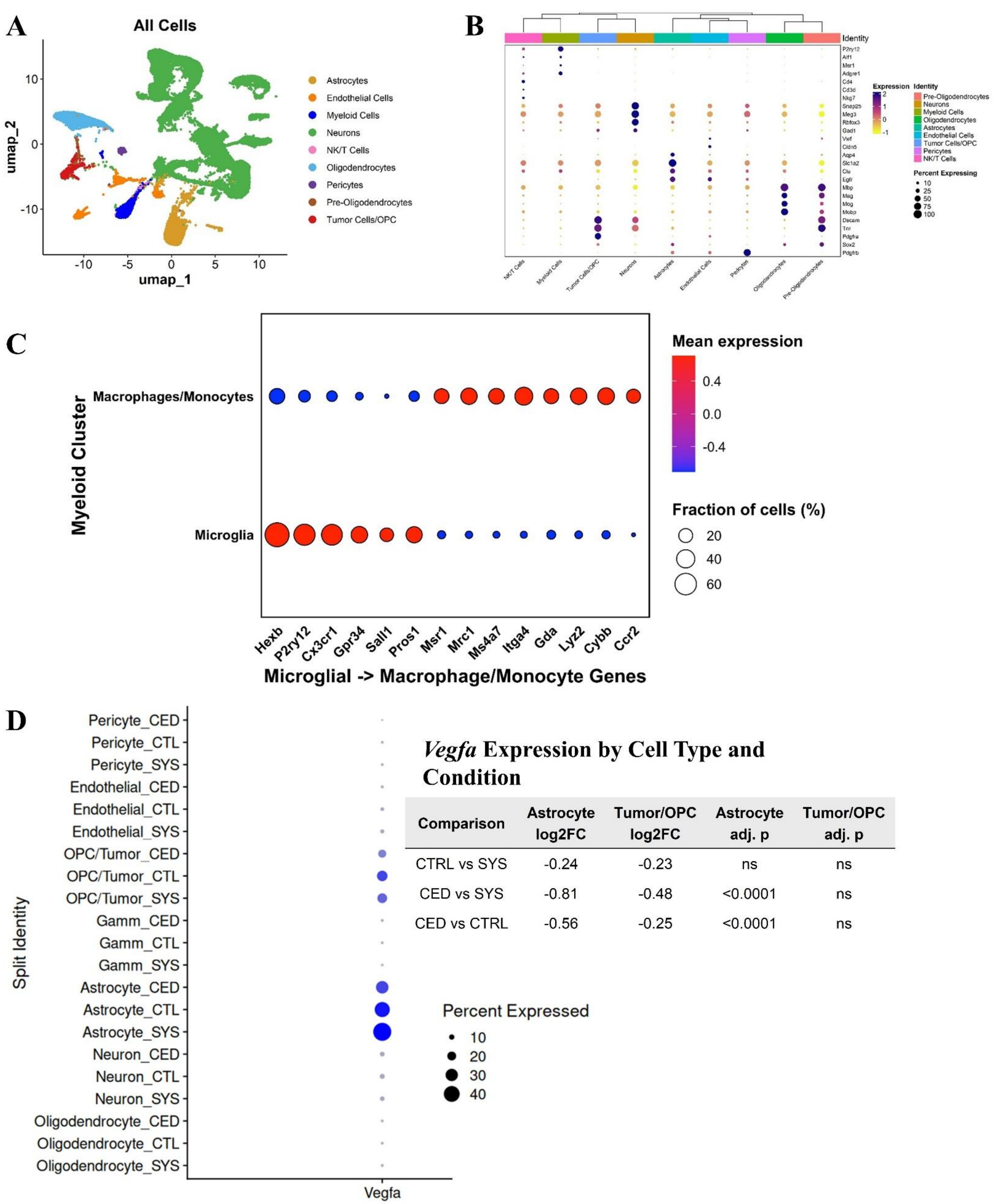

Vegfa **Expression by Cell Type and Condition**

Comparison	Astrocyte log2FC	Tumor/OPC log2FC	Astrocyte adj. p	Tumor/OPC adj. p
CTRL vs SYS	-0.24	-0.23	ns	ns
CED vs SYS	-0.81	-0.48	<0.0001	ns
CED vs CTRL	-0.56	-0.25	<0.0001	ns

Supplementary Figure 3: **A** – Major cell types assigned by SingleR. **B** – Dot plot of canonical marker genes present in major cell lineages. **C** – Microglial and macrophage gene expression across myeloid nuclei using canonical genes. **D –** Vegfa expression by cell type.

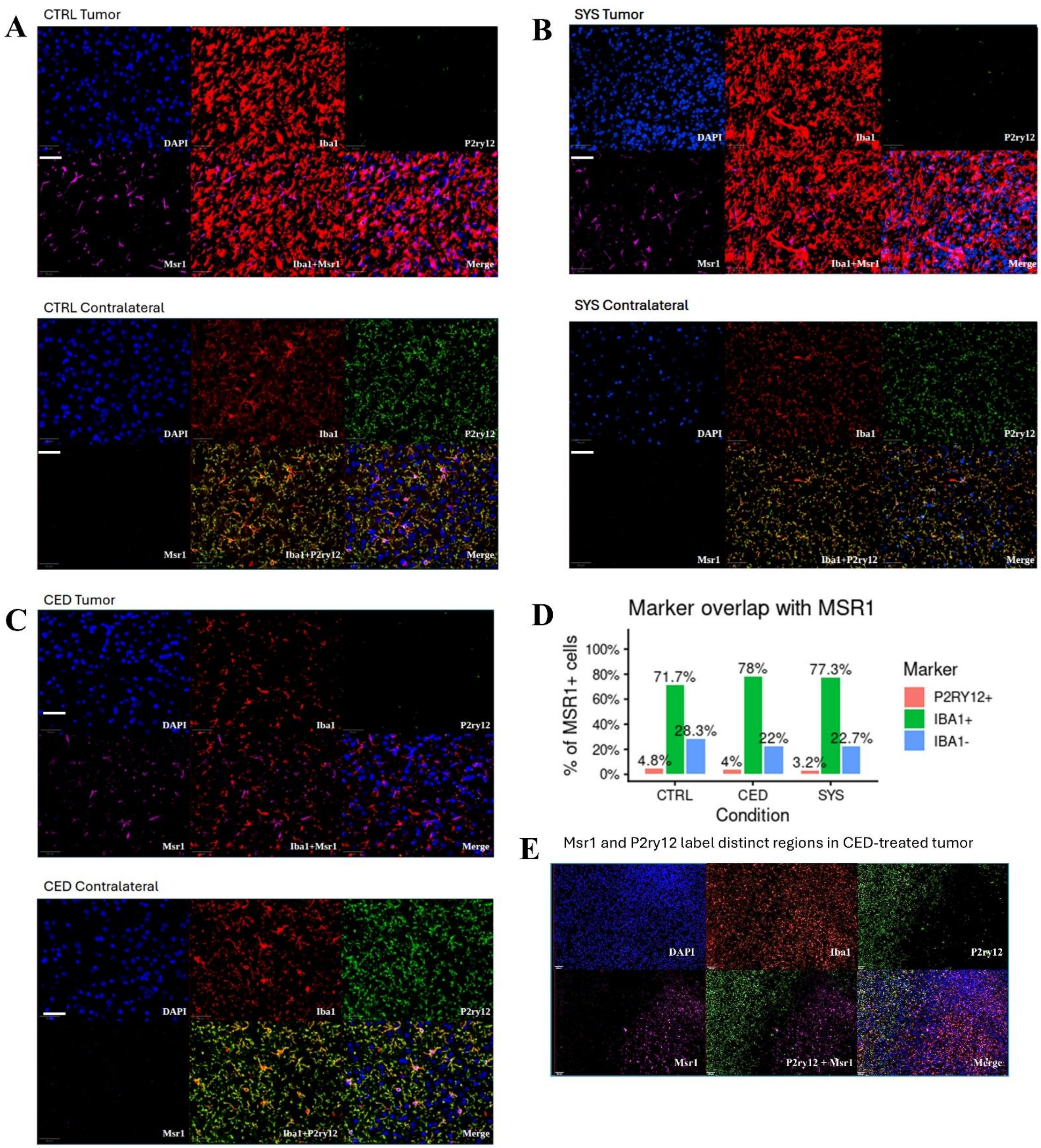

Supplementary Figure 4: A-C – Representative immunofluorescence images of Iba1, Msr1, and P2ry12 in the tumor and contralateral hemisphere of APCL tumor-bearing mice under control, systemic dexamethasone (10 mg/kg/day i.p.), CED-dexamethasone (100 ng/µl in 1007D Alzet pump) treatment conditions, respectively. White bar represents 50 µm. **D** – Quantification from one representative sample per condition showed that Iba1 labeling was present in the majority of Msr1+ cells, supporting their myeloid identity. In contrast, P2ry12 labeling among Msr1+ cells was rare, consistent with these markers identifying largely distinct populations. **E** – Representative image from a CED-dexamethasone-treated mouse demonstrates that Msr1 and P2ry12 label spatially distinct regions, with Msr1 concentrated in the tumor core and P2ry12 localized outside the tumor. White bar represents 100 µm.

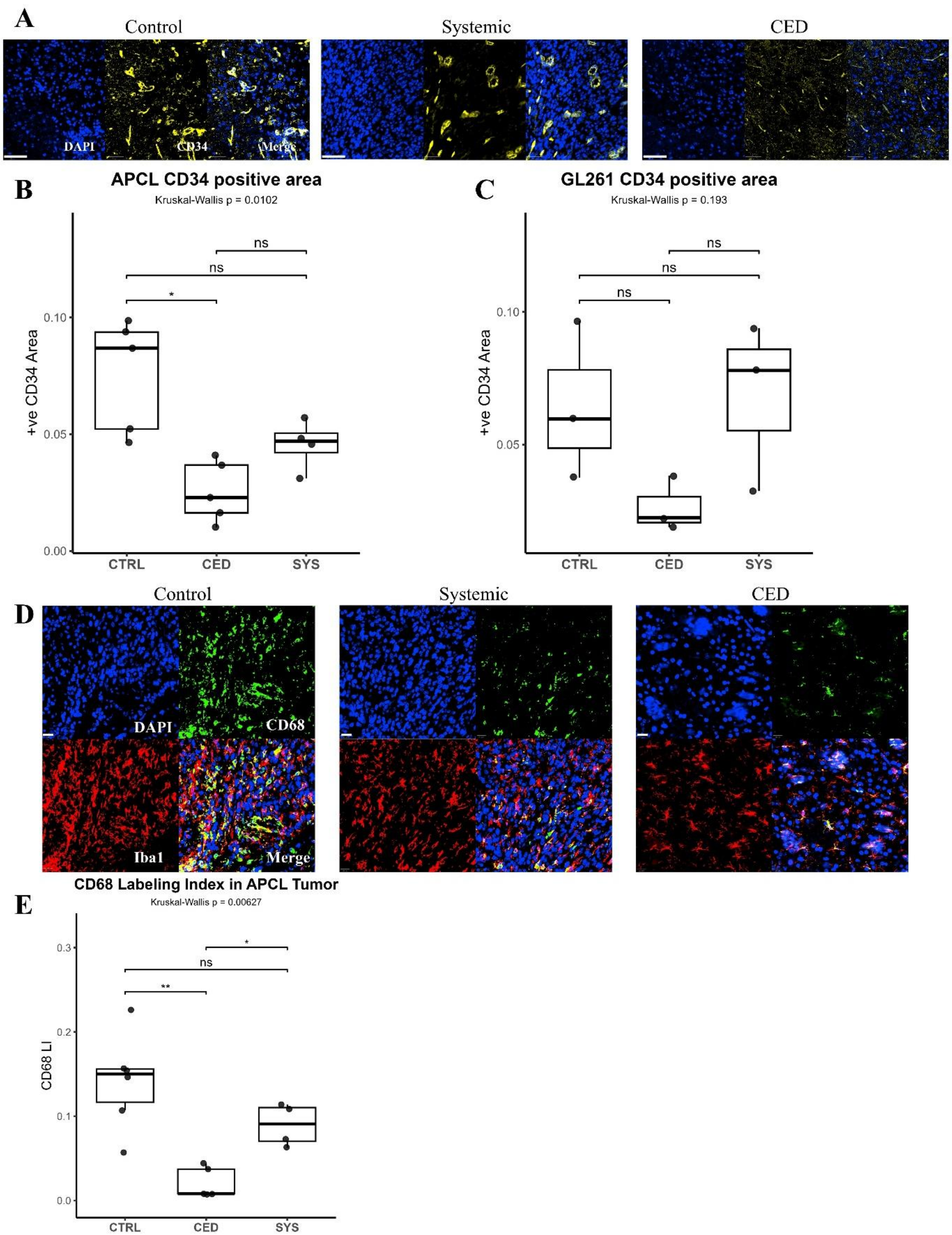

Supplementary Figure 5: **A** – Representative immunofluorescence images showing CD68 and CD34 in the tumor core of GL261 tumor-bearing mice. White bar represents 20 µm. **B** – Both systemic and CED dexamethasone led to a significant reduction in CD68-positive cells compared with control (CTRL vs SYS, $p = 0.0139$; CTRL vs CED, $p = 0.0228$), with no significant difference between the systemic and CED groups (SYS vs CED, $p = 0.658$). **C** – Both systemic and CED dexamethasone also led to a significant reduction in CD34-positive staining area normalized to cellularity compared with control (CTRL vs SYS, $p = 0.0134$; CTRL vs CED, $p = 0.0143$), with no significant difference between the systemic and CED groups (SYS vs CED, $p = 0.527$). Statistical testing was performed using a Kruskal–Wallis test with post-hoc pairwise Welch’s t-tests (* = $p < 0.05$; ** = $p < 0.01$, *** = $p < 0.001$, **** = $p < 0.0001$).

A

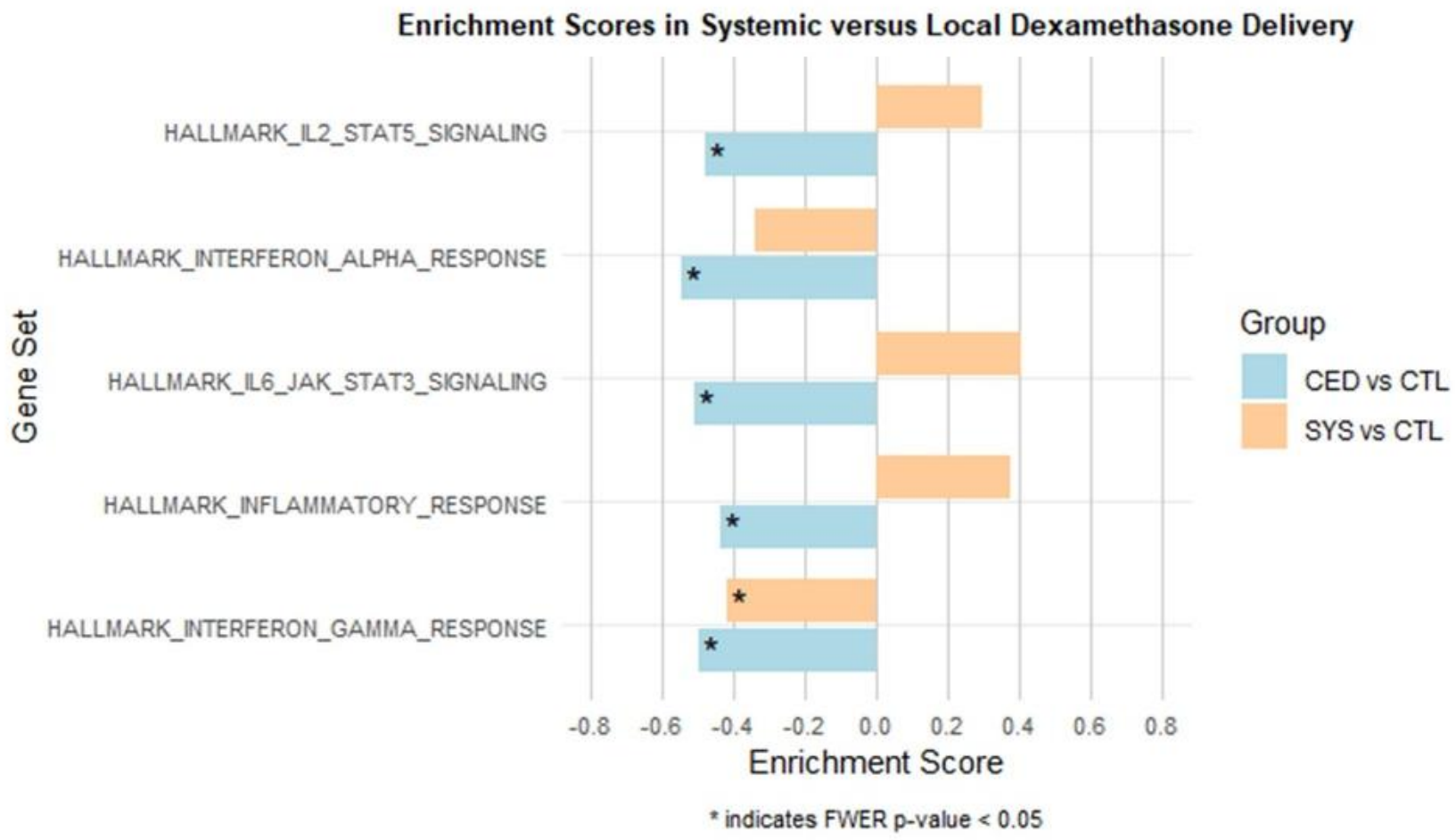

B Programs in Mouse Glioma Myeloid Cells Treated with CTRL vs CED and CTL vs SYS Dexamethasone

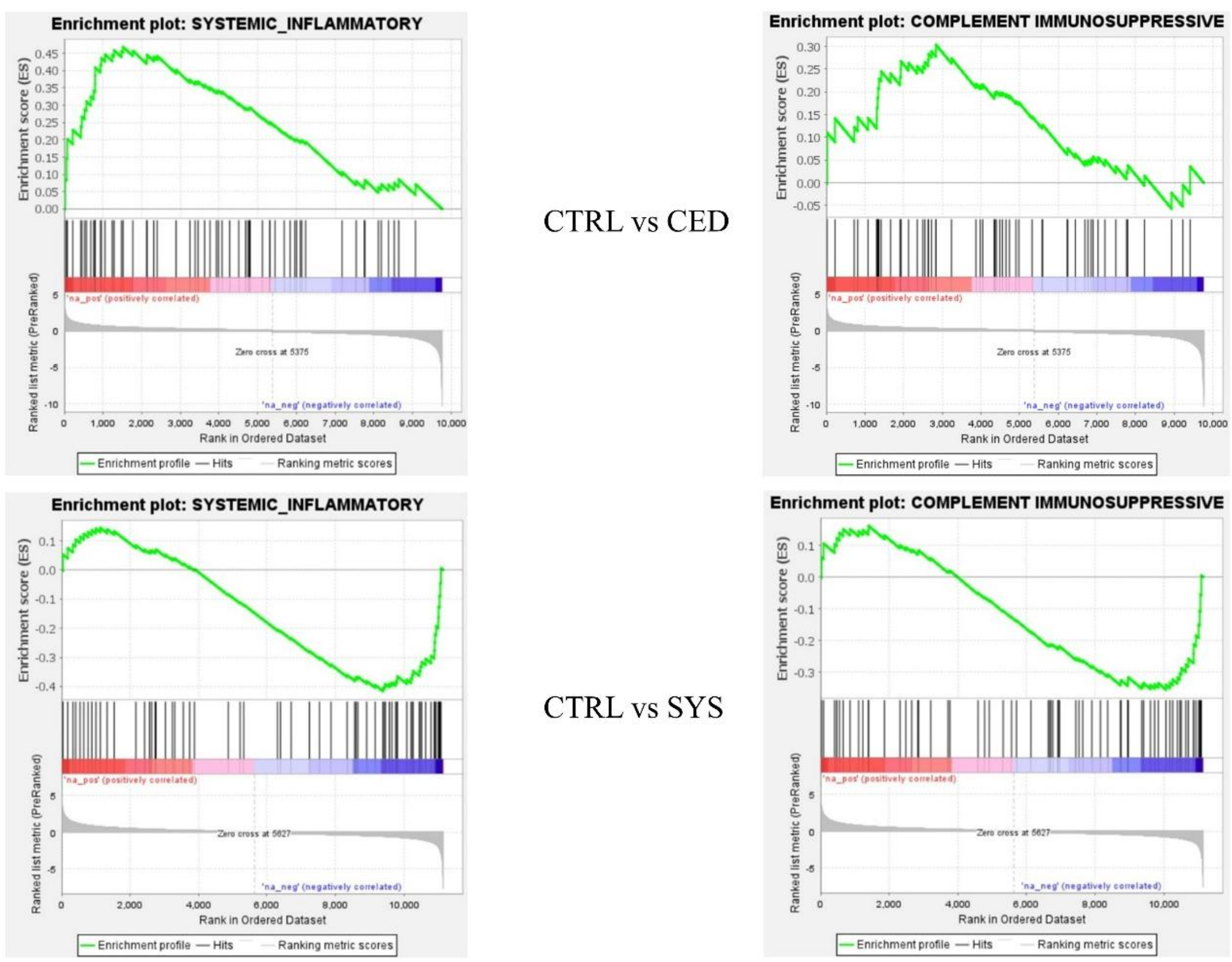

Supplementary Figure 6: **A** – GSEA enrichment scores for select Hallmark inflammatory pathways. Interferon response pathways moved in the same direction, regardless of delivery method, while other inflammatory gene sets moved in opposite directions. Local delivery appears to be more uniformly anti-inflammatory than once daily systemic delivery. **B** – The "complement immunosuppressive" and "systemic inflammatory" pathways from Miller et al. were differentially regulated in the local vs systemic delivery groups. Local delivery led to a NES of -1.39 whereas systemic delivery had a NES of +1.21 in the "complement immunosuppressive" gene set. Similarly, local delivery led to a NES of -1.59 whereas systemic delivery had a NES of +1.91 in the "systemic inflammatory" gene set.c

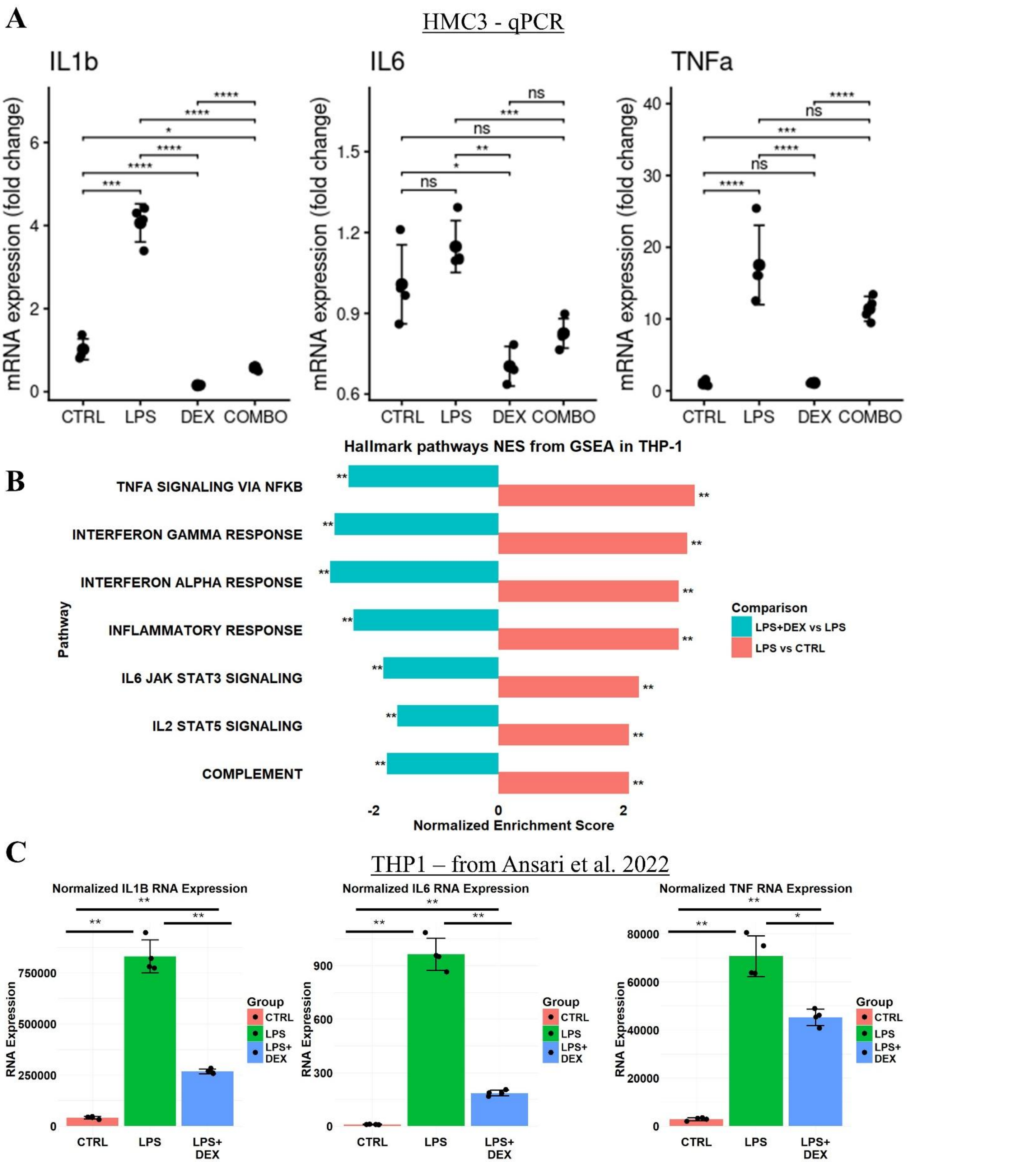

Supplementary Figure 7: **A** – qPCR for *IL1b, IL6,* and *TNFa* expression in human HMC3 microglia treated with vehicle, LPS, dexamethasone, or LPS+dexamethasone. **B** – LPS induced a broad upregulation of Hallmark inflammatory genes in THP-1 cells, which were relatively downregulated when cells were treated with a combination of LPS+Dex. **C** – Normalized RNA expression for IL-1β, IL6, and TNFα. Statistical testing was performed using a Kruskal–Wallis test with post-hoc pairwise Welch's t-tests (* = $p < 0.05$; ** = $p < 0.01$, *** = $p < 0.001$, **** = $p < 0.0001$)

Supplementary Table 1

Nominal dexamethasone concentration was 100 ng/µl.

Days at 37C	Dexamethasone concentration (ng/µl)
1	93.99
3	91.02
4	91.05
5	91.11
6	95.76
7	92.42
8	92.21
10	94.42

Supplementary Table 1: Dexamethasone remains stable at physiologic conditions for up to 10 days.

Supplementary Table 2

**Gene Name**
ABHD12
AKAP13
APBB1IP
ASAP1
BASP1
BIN2
CTSS
CD2
CD74
CMSS1
C1QC
CX3CR1
CST3
DOCK8
DAPP1
FGD2
GPR34
GZMA
HPGDS
ITGA6
IFI27
IL2RA
LAIR1
LY86
MALAT1
MEF2C
NAV2
NAV3
NR3C1
PTN
PLXDC2
PENK
RAD51B
ARHGAP45
SCIN
SLAMF7
SUSD3
TSPAN8
TREM2
UNC93B1

Supplementary Table 2: Top downregulated genes in CED-treated, myeloid nuclei in the snRNA data compared to control. This gene set was used to assess the prognostic importance of suppressing an inflammatory transcriptional signature by correlating survival to the expression of these genes in the TCGA and CGGA datasets.

Supplementary Table 3

Gene Rank	Gene	avg_log2FC	p_val_adj
1	Fkbp5	-1.458	1.71E-72
2	Mir99ahg	1.925	5.98E-52
3	Ccnd3	-1.371	5.58E-51
4	Nav2	2.094	1.70E-47
5	Slc9a9	1.267	2.25E-37
6	Zbtb16	-1.815	6.84E-37
7	Gcnt2	-1.985	1.50E-31
8	Tanc2	0.957	2.17E-31
9	Arl15	-1.208	3.57E-31
10	Plxdc2	1.205	9.57E-31
11	Tmcc3	0.918	2.77E-29
12	Cpeb2	-1.230	3.18E-23
13	Csmd3	1.839	1.31E-22
14	Fn1	-3.367	5.00E-22
15	Rnf169	-0.829	9.45E-22
16	Klf13	-1.074	1.14E-20
17	Ap1s2	-1.486	3.06E-20
18	Lrmda	0.938	1.73E-19
19	Ctsl	-1.425	3.79E-18
20	Slc24a3	-1.213	7.69E-18
21	Il4ra	-1.929	1.07E-17
22	C5ar1	-2.176	1.39E-17
23	Inpp4b	0.867	5.36E-17
24	Ssh2	-0.560	7.43E-17
25	Rab27a	-1.426	2.03E-16
26	Kdm7a	-0.997	8.61E-16
27	Arhgap45	-0.712	1.29E-15
28	Plxna4	1.460	1.94E-15
29	Lifr	-1.130	2.09E-15
30	Il1r2	-5.533	2.44E-15
31	Pik3ip1	-2.348	2.56E-15
32	Mcl1	-1.267	1.49E-14
33	Fchsd2	1.180	1.88E-14
34	Chil3	-5.383	6.98E-14
35	Cebpb	-1.972	7.65E-14
36	Slc38a2	-1.035	1.30E-13
37	Snx29	0.868	1.82E-13
38	Large1	1.184	2.87E-13
39	Rnf180	1.416	3.88E-13
40	Cd300lf	-1.376	4.27E-13
41	Peli2	-0.846	7.77E-13
42	Spp1	-3.740	1.24E-12
43	Fcgr3	-1.288	1.25E-12
44	Igf1r	-0.999	3.62E-12
45	Ptpn1	-0.863	4.42E-12
46	Cd244a	-1.378	1.04E-11
47	Picalm	-0.566	1.38E-11
48	Arhgap24	-0.882	4.38E-11
49	Ctss	-0.678	4.75E-11
50	Slc39a14	-2.048	5.70E-11
51	Mir142hg	-1.058	6.40E-11
52	Cept1	-0.854	2.23E-10
53	Alox5ap	-1.734	3.09E-10
54	Tpd52	-0.936	3.14E-10
55	Rin3	-0.861	3.72E-10
56	Frmd4a	0.841	3.74E-10
57	Socs3	-3.058	3.99E-10
58	Aoah	2.036	4.11E-10
59	Ms4a6c	-1.392	5.23E-10
60	Per1	-2.289	8.56E-10
61	Maml3	-0.649	1.28E-09
62	Herc4	-0.760	2.11E-09
63	Mafb	-0.986	2.17E-09
64	Osbpl9	-0.939	2.23E-09
65	Mmp8	-4.979	2.25E-09
66	Gm13710	3.122	2.58E-09
67	Hp	-3.319	3.13E-09
68	Gm10790	1.147	3.34E-09
69	C030034L19Rik	-1.619	3.34E-09
70	Slc8a1	0.464	3.58E-09
71	Fcer1g	-1.167	3.79E-09
72	Cdk8	-0.739	4.02E-09
73	Gsr	-1.386	4.81E-09
74	Tmem154	-2.193	5.45E-09
75	Tmem87b	-0.734	7.64E-09
76	Notch4	-1.699	7.88E-09
77	Sgip1	-1.113	9.68E-09
78	Ripor2	-0.983	9.93E-09
79	Tsc22d3	-0.800	1.60E-08
80	Gda	-2.186	1.96E-08
81	Ucp2	-1.168	2.85E-08
82	Arrb2	-0.765	3.35E-08
83	Dhx9	-0.744	3.45E-08
84	Ddi2	-0.669	4.40E-08
85	Ms4a8a	-3.338	5.45E-08
86	Camk1d	-0.394	6.19E-08
87	Srgap2	0.516	7.29E-08
88	Stab1	-0.709	7.99E-08
89	Ctsd	-0.694	1.00E-07
90	Rassf2	-0.701	1.01E-07
91	Cd180	1.433	1.20E-07
92	Man2a1	-0.627	1.35E-07
93	Nfam1	-0.891	1.42E-07
94	F10	-3.302	1.50E-07
95	Inf2	-1.580	1.74E-07
96	Ston1	-1.567	1.78E-07
97	Ms4a4a	-1.544	1.80E-07
98	Fxyd5	-1.902	1.91E-07
99	Rcsd1	-0.710	1.96E-07
100	Sla	-0.693	2.02E-07
101	Igsf6	-1.179	2.46E-07
102	Ms4a6d	-1.102	2.67E-07
103	Clk4	-0.897	2.69E-07
104	Srgn	-2.706	2.78E-07
105	Cmss1	-0.595	2.94E-07
106	Ccr2	-2.101	3.08E-07
107	Ccdc192	3.420	3.52E-07
108	Snx1	-0.901	4.01E-07
109	Bin2	-0.650	4.78E-07
110	Sbno2	-0.871	6.93E-07
111	Arpc1b	-1.024	8.06E-07
112	Frrs1	-0.746	8.39E-07
113	S100a10	-2.911	9.63E-07
114	Cast	-1.265	9.81E-07
115	Pde2a	-0.873	1.06E-06
116	Pag1	-0.463	1.13E-06
117	Emilin2	-2.439	1.23E-06
118	Ctsa	-0.920	1.25E-06
119	Cep152	-1.017	1.27E-06
120	Mmp19	-4.773	1.30E-06
121	Serp1	-1.365	1.40E-06
122	Hnrnpf	-0.905	1.41E-06
123	Il17ra	-1.070	1.49E-06
124	Gphn	-0.582	1.62E-06
125	Rbm47	-0.374	1.63E-06
126	Slc43a2	-0.640	1.63E-06
127	Fcgr2b	-0.960	1.67E-06
128	H2-D1	-0.914	1.91E-06
129	Tcn2	-1.216	2.34E-06
130	Nufip2	-0.582	2.70E-06
131	Ttc28	0.881	2.71E-06
132	Cux1	-0.519	2.91E-06
133	Srsf7	-0.705	3.03E-06
134	Ophn1	0.556	3.10E-06
135	Washc2	-0.805	3.15E-06
136	Tgfbi	-2.264	3.26E-06
137	Mia2	-0.620	3.38E-06
138	Ly86	0.759	3.46E-06
139	Atrnl1	-1.001	3.81E-06
140	Mafg	-0.726	3.92E-06
141	Comt	-0.513	4.47E-06
142	Diaph1	-0.993	4.54E-06
143	Ogfrl1	-0.706	5.93E-06
144	Mt2	-1.067	5.97E-06
145	Tbc1d15	-1.084	6.01E-06
146	Itga9	1.430	7.18E-06
147	Polg	-0.856	7.25E-06
148	Hmox1	-1.613	8.18E-06
149	Rbm26	-0.564	8.25E-06
150	Clec4d	-3.201	8.28E-06
151	Cadm1	0.950	8.28E-06
152	Gm19951	-0.770	8.46E-06
153	Tet2	-0.637	8.82E-06
154	Qki	0.440	9.29E-06
155	Swt1	-0.837	9.42E-06
156	Nfkbia	-1.240	9.51E-06
157	Tg	-2.710	9.63E-06
158	Ccr1	-2.508	1.01E-05
159	Hdac4	-0.897	1.05E-05
160	Dok3	-1.331	1.06E-05
161	Fndc3b	-0.642	1.13E-05
162	Il15ra	-0.956	1.14E-05
163	Stat3	-0.684	1.15E-05
164	Gm56663	-4.042	1.23E-05
165	Vsir	-0.501	1.24E-05
166	Meg3	0.756	1.31E-05
167	Ptprm	1.180	1.44E-05
168	Zdhhc9	-1.471	1.63E-05
169	2210408F21Rik	-0.890	2.11E-05
170	Klhl24	-0.909	2.12E-05
171	Thbs1	-4.183	2.19E-05
172	Fbxl20	-0.565	2.21E-05
173	Filip1l	-0.531	2.22E-05
174	Jdp2	-1.082	2.29E-05
175	Pgd	-1.096	2.31E-05
176	Msrb1	-2.143	2.75E-05
177	Btg1	-1.136	2.91E-05
178	Ifitm3	-2.200	2.99E-05
179	Otub1	-1.988	3.05E-05
180	Lpcat2	0.796	3.18E-05
181	Tm6sf1	-0.426	3.22E-05
182	Zfp697	-0.958	3.29E-05
183	Tyrobp	-1.616	3.96E-05
184	Ltb4r1	-4.649	3.97E-05
185	Abhd15	-0.786	4.00E-05
186	Parp4	-0.982	4.06E-05
187	Calr	-0.981	4.31E-05
188	Ddit4	-1.367	4.83E-05
189	Man2b1	-0.625	5.01E-05
190	Map3k8	-0.896	5.12E-05
191	Ifngr1	-0.527	5.36E-05
192	Dmxl2	-0.927	6.06E-05
193	Zfp36l2	-0.826	6.06E-05
194	Osbpl11	-0.528	6.43E-05
195	Svil	-0.919	6.46E-05
196	Npnt	2.890	6.90E-05
197	Tpp2	-0.678	6.92E-05
198	Klhl2	-0.931	6.93E-05
199	Adgre1	1.278	6.99E-05
200	Dusp3	-0.847	7.26E-05
201	Cdk14	-0.505	7.61E-05
202	Kcnma1	1.286	8.05E-05
203	Neat1	-1.254	8.33E-05
204	Dram2	-0.462	8.71E-05
205	Sell	-4.102	1.07E-04
206	Atp8b4	-1.301	1.14E-04
207	Pkn1	-0.557	1.29E-04
208	Jak1	-0.496	1.32E-04
209	Klf9	-0.966	1.35E-04
210	Pfkfb3	-0.707	1.37E-04
211	Tspan5	-0.694	1.38E-04
212	Wdr26	-0.487	1.41E-04
213	Chd9	0.496	1.55E-04
214	S100a11	-3.531	1.61E-04
215	Knop1	-0.751	1.67E-04
216	Hif3a	-1.886	1.76E-04
217	Vcan	-2.863	1.83E-04
218	Ppp1r10	-0.677	1.84E-04
219	Tmod1	-1.093	1.90E-04
220	Stat1	1.301	2.09E-04
221	Elmo1	0.272	2.11E-04
222	Il6ra	-0.540	2.18E-04
223	Rnf149	-0.817	2.31E-04
224	H2-K1	-0.738	2.45E-04
225	Sirpa	0.665	2.49E-04
226	Aopep	-0.558	2.85E-04
227	Iigp1	2.981	2.87E-04
228	Hspa5	-0.683	3.00E-04
229	Slc30a5	-0.926	3.17E-04
230	Grk6	-1.174	3.60E-04
231	Slc2a1	-1.582	3.76E-04
232	Shisa5	-1.500	4.24E-04
233	Stx5a	-0.674	4.35E-04
234	Mt1	-0.614	4.45E-04
235	Smpdl3a	-1.882	5.07E-04
236	Pkm	-0.582	5.10E-04
237	Gpi1	-0.619	5.25E-04
238	Grn	-0.691	5.39E-04
239	Sdc4	-1.265	5.69E-04
240	Esco1	-0.568	5.79E-04
241	Zbed6	-0.852	5.81E-04
242	Ube2h	-0.458	5.91E-04
243	Atp8a1	-0.464	5.93E-04
244	Slc2a3	-0.601	6.08E-04
245	Csf2rb	-0.854	6.16E-04
246	Sort1	-0.489	6.19E-04
247	Tenm2	1.830	6.58E-04
248	Hipk1	-0.756	6.70E-04
249	S100a6	-2.519	6.76E-04
250	Alpk1	-1.089	6.78E-04
251	Sipa1	-0.398	7.02E-04
252	Ldlrad4	0.490	7.08E-04
253	1600014C10Rik	-1.053	7.14E-04
254	Rassf3	-0.556	7.85E-04
255	Arg1	-3.250	8.33E-04
256	Pdk1	-0.476	8.52E-04
257	Nisch	-0.516	9.03E-04
258	Hbp1	-0.609	9.09E-04
259	Xbp1	-0.998	9.32E-04
260	Gpr146	-1.018	9.65E-04
261	Lrp1b	1.869	9.74E-04
262	Prkag2	-0.776	9.89E-04
263	Apoc2	-3.869	1.03E-03
264	Slfn2	-1.161	1.10E-03
265	Rc3h1	-0.576	1.14E-03
266	Fbxl5	-0.850	1.15E-03
267	Itgb2	-0.662	1.17E-03
268	Plet1os	-2.536	1.20E-03
269	Rnpep	-0.640	1.25E-03
270	Cd53	-0.712	1.27E-03

271	Apobec1	-0.415	1.28E-03
272	Tmem156	-1.363	1.34E-03
273	Mast3	-0.700	1.36E-03
274	Npepps	-0.667	1.37E-03
275	Hacd2	-0.731	1.37E-03
276	Scarb1	-0.870	1.38E-03
277	Gm33100	-1.164	1.41E-03
278	Atrn	-0.859	1.49E-03
279	Dpp8	-0.595	1.53E-03
280	Trappc14	-0.586	1.54E-03
281	Malat1	0.129	1.67E-03
282	Pygl	-1.624	1.68E-03
283	Ostf1	-0.543	1.68E-03
284	Tiparp	-1.532	1.73E-03
285	Clk1	-0.440	1.77E-03
286	Pirb	-1.519	1.79E-03
287	Fth1	0.136	1.79E-03
288	Lars2	-0.562	1.81E-03
289	Chaserr	-0.494	1.82E-03
290	Rbm5	-0.434	1.84E-03
291	Prelid1	-1.556	1.87E-03
292	Emb	-1.778	1.88E-03
293	Eed	-0.637	1.91E-03
294	Ctsz	-0.526	1.94E-03
295	Ibtk	-1.320	1.96E-03
296	Ctsh	-0.477	1.99E-03
297	Maml2	0.548	2.02E-03
298	Cyba	-1.058	2.10E-03
299	Macir	-0.929	2.13E-03
300	B4galt1	-0.365	2.14E-03
301	Tmbim6	-0.396	2.16E-03
302	Baz1a	-0.417	2.26E-03
303	Stk24	-0.598	2.30E-03
304	Plaur	-1.409	2.35E-03
305	Arhgef18	-0.905	2.42E-03
306	Zfc3h1	-0.461	2.46E-03
307	Ahnak	-1.426	2.48E-03
308	Wipf3	-1.339	2.50E-03
309	Cd63	-0.997	2.51E-03
310	Nrxn3	0.653	2.61E-03
311	Snx18	-0.709	2.67E-03
312	Rab21	-0.547	2.77E-03
313	Sorl1	-0.551	2.82E-03
314	Pak2	-0.417	2.87E-03
315	Tns3	0.658	2.89E-03
316	Cd68	-1.099	2.93E-03
317	Il18rap	-2.097	2.97E-03
318	Arhgap30	-0.513	3.01E-03
319	Selplg	-0.478	3.33E-03
320	Sirpb1c	-2.454	3.39E-03
321	Capg	-1.971	3.56E-03
322	Gnai2	-0.750	3.73E-03
323	Smad4	-0.511	3.81E-03
324	Irak4	-0.652	3.82E-03
325	Dock2	0.432	3.87E-03
326	Iqgap1	-0.779	4.00E-03
327	Prpf40a	-0.585	4.04E-03
328	Csad	-0.759	4.21E-03
329	Laptm5	-0.525	4.39E-03
330	Cebpg	-0.569	4.40E-03
331	Elf4	-0.461	4.56E-03
332	Tcp11l2	-1.014	4.68E-03
333	Mau2	-0.459	4.85E-03
334	Arpc2	-0.396	5.03E-03
335	Plod1	-0.616	5.07E-03
336	Rsrp1	-0.437	5.14E-03
337	4930447J18Rik	-4.951	5.16E-03
338	Coro1a	-0.854	5.19E-03
339	Napsa	-3.087	5.34E-03
340	Mlph	-0.629	5.39E-03
341	Adamtsl4	-1.598	5.53E-03
342	Fmnl1	-0.630	5.55E-03
343	Rnf145	-0.674	5.59E-03
344	Coro2a	-0.482	5.77E-03
345	Dapp1	-0.412	5.80E-03
346	Slc16a6	-0.651	5.86E-03
347	Nfil3	-2.408	6.08E-03
348	Vgll4	-0.538	6.17E-03
349	Gm48633	-7.700	6.21E-03
350	Ms4a4c	-1.720	6.28E-03
351	Kmt2e	-0.332	6.50E-03
352	Txnip	-0.959	6.52E-03
353	Zfp740	-0.636	6.54E-03
354	Fhit	1.188	6.65E-03
355	Flot1	-1.242	6.70E-03
356	Rab5if	-0.982	7.16E-03
357	Snrnp70	-0.380	7.29E-03
358	Rpgrip1	-0.916	7.31E-03
359	Ifi209	-1.350	7.59E-03
360	Cebpd	-1.447	7.69E-03
361	Pdia3	-0.434	7.84E-03
362	Abcg1	-0.487	7.91E-03
363	Epb41l2	0.495	8.11E-03
364	Rab32	-0.999	8.29E-03
365	Tars2	-1.213	8.49E-03
366	Nipa2	-0.563	8.52E-03
367	Abca6	-3.576	8.67E-03
368	Sell1	-0.521	8.69E-03
369	Stk17b	-0.755	8.80E-03
370	Pnkp	-1.741	8.81E-03
371	C1qa	-0.591	9.08E-03
372	Fes	-0.825	9.26E-03
373	Bst1	-2.790	9.32E-03
374	Agl	-0.747	9.41E-03
375	Setx	-0.703	9.42E-03
376	Aff1	-0.447	9.51E-03
377	Gm46224	-0.607	9.94E-03
378	Gcnt1	-0.494	9.94E-03
379	Tent5a	-0.520	1.02E-02
380	Ecm1	-1.752	1.06E-02
381	Trf	-0.419	1.07E-02
382	Ms4a6b	-0.430	1.10E-02
383	Faf2	-0.853	1.12E-02
384	Lrp10	-0.859	1.14E-02
385	Ppard	-0.637	1.15E-02
386	Ypel3	-0.412	1.16E-02
387	Anxa2	-2.047	1.16E-02
388	Prdm2	-0.492	1.19E-02
389	Mlxip	-0.389	1.19E-02
390	Tut7	-0.418	1.19E-02
v391	Phf3	-0.384	1.27E-02
392	Sult1a1	-0.629	1.31E-02
393	U2af1	-0.727	1.39E-02
394	Uhrf1bp1l	-0.736	1.40E-02
395	Eif4ebp2	-0.655	1.45E-02
396	Tspan18	2.603	1.64E-02
397	Ly6c2	-3.861	1.65E-02
398	Wfdc17	-2.947	1.69E-02
399	Bclaf1	-0.340	1.73E-02
400	Lrpap1	-0.677	1.81E-02
401	Rmdn1	-0.510	1.82E-02
402	Arpc4	-0.738	1.83E-02
403	Bank1	1.307	1.86E-02
404	Ssbp1	-0.531	1.89E-02
405	Ifi30	-1.402	1.91E-02
406	Slfn1	-2.085	2.04E-02
407	Gm57206	-0.849	2.05E-02
408	Atp1a1	-0.498	2.05E-02
409	Ifitm2	-2.759	2.07E-02
410	Tgfbr1	0.326	2.16E-02
411	Sfxn5	-1.062	2.21E-02
412	Cyrib	-0.289	2.22E-02
413	Ripk2	-0.608	2.25E-02
414	Fam168a	-0.442	2.28E-02
415	Hnrnpll	-0.767	2.29E-02
416	Grid2	1.976	2.29E-02
417	Syk	-0.481	2.30E-02
418	Mdga2	2.059	2.30E-02
419	Adam9	-0.878	2.31E-02
420	Calhm2	-0.930	2.34E-02
421	Ccar1	-0.539	2.36E-02
422	AB124611	-0.940	2.38E-02
423	Saa3	0.235	2.51E-02
424	Psmd12	-0.705	2.53E-02
425	Tnfsf13	-1.848	2.53E-02
426	Uckl1	-0.873	2.57E-02
427	Adam8	-2.150	2.68E-02
428	Ptprj	0.392	2.71E-02
429	Peli1	0.759	2.72E-02
430	Pitpna	-0.469	2.95E-02
431	Pfn1	-0.855	2.97E-02
432	Patl1	-0.695	2.98E-02
433	Mon2	-0.438	3.02E-02
434	Rbms1	-0.431	3.02E-02
435	Ppan	-2.345	3.15E-02
436	Arl6ip1	-0.397	3.34E-02
437	Adssl1	-1.916	3.36E-02
438	Cfh	-0.575	3.36E-02
439	D530033B14Rik	-1.858	3.37E-02
440	Hmg20b	-0.928	3.48E-02
441	Gm22146	-1.265	3.52E-02
442	Slc38a1	-0.361	3.61E-02
443	Brd8	-0.409	3.63E-02
444	Hnrnpdl	-0.458	3.92E-02
445	Serinc3	-0.327	3.93E-02
446	Ifi207	-0.692	3.96E-02
447	Sdcbp	-0.417	3.96E-02
448	Scamp1	-0.704	4.00E-02
449	Nars	-0.544	4.00E-02
450	Crot	-0.675	4.13E-02
451	Ncf1	-0.369	4.15E-02
452	Dlg2	1.063	4.16E-02
453	Crk	-0.545	4.28E-02
454	Ero1b	-1.064	4.28E-02
455	Scyl3	-0.902	4.44E-02
456	Pign	-0.405	4.49E-02
457	Nbr1	-0.376	4.60E-02
458	Sp3	-0.499	4.71E-02
459	Acp5	-3.136	4.82E-02
460	3110082I17Rik	-0.465	4.86E-02
461	C1qc	-0.427	4.86E-02
462	Eif5	-0.532	4.92E-02
463	Secisbp2	-0.501	4.99E-02

Supplementary Table 3: Differentially expressed genes in myeloid cells between mice treated with CED-dexamethasone and systemic dexamethasone by snRNA-sequencing.
